# Animal carcass burial management: implications for sustainable biochar use

**DOI:** 10.1186/s13765-021-00652-z

**Published:** 2021-12-22

**Authors:** Meththika Vithanage, S. S. Mayakaduwage, Viraj Gunarathne, Anushka Upamali Rajapaksha, Mahtab Ahmad, Adel Abduljabbar, Adel Usman, Mohammad I. Al-Wabel, James A. Ippolito, Yong Sik Ok

**Affiliations:** 1 Ecosphere Resilience Research Centre, Faculty of Applied Sciences, University of Sri Jayewardenepura, Nugegoda, 10250 Sri Lanka; 2 School of Agriculture, Food and Wine, University of Adelaide, Adelaide, Australia; 3 Department of Environmental Sciences, Faculty of Biological Sciences, Quaid-i-Azam University, Islamabad, 45320 Pakistan; 4 Industrial Psychology, College of Education, King Saud University, Riyadh, Saudi Arabia; 5 Soil Sciences Department, College of Food and Agricultural Sciences, King Saud University, Riyadh, Saudi Arabia; 6 Department of Soil and Crop Sciences, Colorado State University, Fort Collins, CO USA; 7 Korea Biochar Research Center, APRU Sustainable Waste Management and Division of Environmental Science and Ecological Engineering, Korea University, Seoul, 02841 South Korea

**Keywords:** Pandemic, Soil amendment, Carcass burial, Human corpses, Biochar

## Abstract

This review focuses on existing technologies for carcass and corpse disposal and potential alternative treatment strategies. Furthermore, key issues related to these treatments (e.g., carcass and corpse disposal events, available methods, performances, and limitations) are addressed in conjunction with associated environmental impacts. Simultaneously, various treatment technologies have been evaluated to provide insights into the adsorptive removal of specific pollutants derived from carcass disposal and management. In this regard, it has been proposed that a low-cost pollutant sorbent may be utilized, namely, biochar. Biochar has demonstrated the ability to remove (in)organic pollutants and excess nutrients from soils and waters; thus, we identify possible biochar uses for soil and water remediation at carcass and corpse disposal sites. To date, however, little emphasis has been placed on potential biochar use to manage such disposal sites. We highlight the need for strategic efforts to accurately assess biochar effectiveness when applied towards the remediation of complex pollutants produced and circulated within carcass and corpse burial systems.

**Graphical Abstract:**

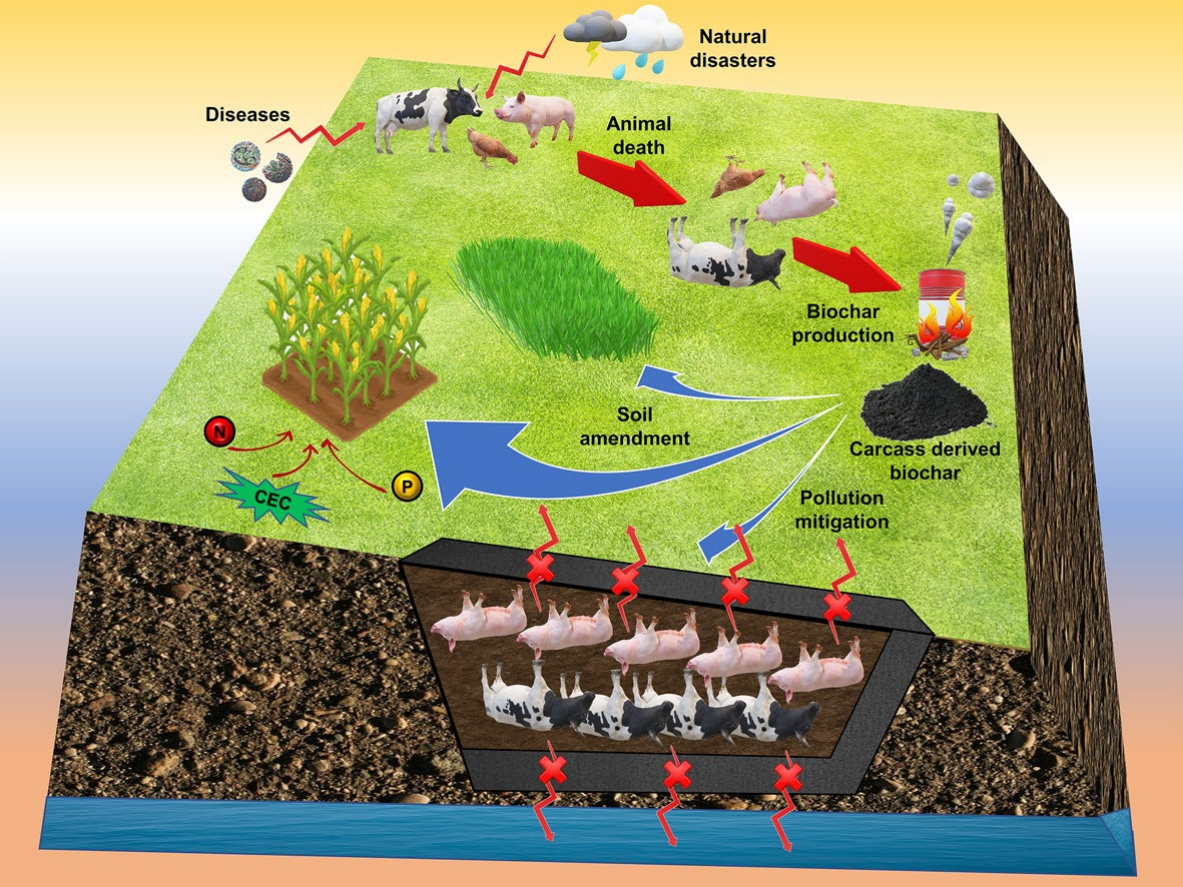

## Introduction

Recently, a growing global concern over the safe and proper disposal of mass carcasses and corpses due to pandemics, epidemics, natural disasters, and wars has arisen. Specifically, at the time of writing, more than 100 countries are facing mass grave or corpse burial issues related to the COVID-19 pandemic. Presently, several countries (e.g., USA, Iran, Italy) are utilizing mass grave burial, while others (e.g., South Africa) are still at an early stage to consider such a disposal means due to rising death tolls from the infectious SARS-CoV-2 contagion. In 2004, one of the most disastrous tsunamis occurred in the Indian Ocean, affecting over 14 countries and killing ~ 230,000 human beings [1]. Mass burial is the only fatality management option following pandemics or natural disasters such as these [2].

Similarly, yet much less notable by the public, animal agricultural operations have identical concerns to those above. Animal agriculture, with a global livestock population of approximately 1.9 × 10^10^ birds and 2.31 × 10^8^ mammals, continuously generates significant carcass volumes that need to be disposed of in a diligent and environmentally responsible manner [3]. For example, the U.S. livestock industry annual production of animal by-products and animal mortality is approximately 2.85 × 10^10^ kg [4]. In Europe, approximately 3.4 × 10^9^ kg of animal carcasses are reported to remain following human consumption [5]. China produces 20 × 10^5^ pig carcasses annually, with this number increasing yearly [6]. The above mentioned numbers, being already considerably high, become greater in the event of disease outbreaks, natural disasters, or other accidents. According to recent data, 32 million livestock units (LSU) die annually due to foot-and-mouth disease (FMD) [7]. In Korea alone, nearly 3.4 × 10^6^ of swine and bovine were slaughtered and buried between 2010 to 2011 due to FMD [8, 9]. In Russia, from 2007 to 2012, over 6.0 × 10^5^ pigs were killed or culled due to swine flu [10]. In 2014, avian influenza caused approximately 7.0 × 10^6^ poultry deaths in Korea [11]. In 2010, extreme weather conditions in Mongolia were accompanied by significant livestock mortality that accounted for ~ 20% of the national herd [12]. The above numbers strongly indicate measures for proper carcass disposal in lieu of environmental safety measures to prevent the spread of disease and contamination of soil and groundwater.

Many authors have reported potential contamination in soil and groundwater systems from mass carcass burial due to organic components (biological oxygen demand (BOD), total organic carbon (TOC), inorganic compounds (e.g., total dissolved solids (TDS), chloride, ammonia, and nitrate), and many other substances (e.g., enteric pathogens, steroid hormones, and antibiotics) [8, 13–15]. In terms of human deaths, the environmental threats posed by mass burial events due to wars, natural disasters or pandemics may be less anticipated than animal carcass disposal events. When human death rates reach historic highs, mass burial often becomes the only viable option [16]. Mass burial events have led to groundwater contamination by hazardous leachate plumes generated by human corpse decomposition [17]. However, the risk of spread of pathogenic organisms through groundwater contamination is of high concern [18]. In terms of animal fatalities, a few studies have focused on mass livestock burial correlations with groundwater contamination, suggesting a need for site-specific contaminant removal technologies [8, 15]. Environmental remediation in the vicinity of mass burial sites should be made via cost-effective methods.

A potential cost-effective tool for addressing environmental remediation of mass burial sites may simply be biochar; biochar has been recognized as an effective, bio-secure, and economically viable approach for addressing the environmental repercussions of animal disposal techniques [19]. Biochar has also been proven to have potential for remediation of pathogen [20], ammonium nitrogen [21], nutrients [22], heavy metals [23], BOD [24], chemical oxygen demand [25], antibiotics [26], and steroid hormone [27] in aqueous/soil media. In this review, we (1) provide a concise and up-to-date overview of carcass management techniques along with their environmental implications, (2) emphasize the significance of biochar use for overcoming carcass management issues, and (3) suggest future research directions.

## Disposal events and technologies for carcasses and corpses

### Mass carcass and corpse disposal events

All animal mortalities, including routine animal mortalities or large animal carcass quantities resulting from hazardous events or disease-related disasters, should be disposed of in a timely, efficient, and safe manner in order to minimize negative environmental impacts. The selected method of disposal may be based on several factors, such as type and quantity of animal carcasses, location, cause of mortality, and economic status [28]. A combination of methods is often required to accomplish carcass disposal, especially during times of significant loss. This may lead to rapid and effective disease eradication. Table 1 summarizes mass carcass and corpse disposal events recorded in different countries, and the disposal methods implemented.

**Table 1:** Carcass and corpse disposal events in the world

Country	Disposal events	Disposal method	Reference
Carcass disposal events			
USA	Routine mortalities	Burial, Composting, Rendering	[11, 32, 36]
	Hurricane Floyd	Burial, Incineration, Composting, Rendering	[13, 34]
	Texas Flood	Burial, Incineration	[34]
	Exotic Newcastle Disease (END)	Burial	[13]
	Severe winter and flood	Burial, Incineration	[13]
	Avian influenza (AI)	Burial	[13]
	A chemical poisoning incident	Burial	[13]
	A fire accident	Burial	[13]
Taiwan	Foot-and-mouth disease (FMD)	Burning, incineration, Rendering	[34, 149]
UK	Bovine spongiform encephalopathy (BSE)	Burial	[36]
	FMD	Burial Rendering Burning Incineration Composting	[13, 14, 36]
	AI	Incineration, Rendering	[150]
Korea	FMD	Burial	[94]
	AI	Burial	[94]
	Routine mortality	Burial, Rendering, Incineration	[11, 94]
Japan	FMD	Burial	[151]
Canada	AI and END	Composting	[152]
Netherlands	FMD and AI	Incineration, Composting	[150]
Hong Kong	AI	Incineration, Composting	[150]
Mass corpse disposal events			
Rwanda	Genocide violence	Burial	[31]
Southeast Poland	World War I and II	Burial	[29]
Britain	World War II	Burial	[153]
Spain	Civil War	Burial	[154]
Haiti	Earthquake	Burial	[155]
Thailand	Tsunami	Burial, Cremation	[30]

### Mass carcass and corpse disposal technologies

The most widely used mass animal carcass and human corpse disposal methods are burial and incineration, that have been used for pandemics, wars and natural catastrophic events [29, 30]. Owing to land scarcity, many countries utilize mass graves for corpse disposal due to aforementioned catastrophic events [31]. However, anaerobic digestion, rendering, alkaline hydrolysis, composting, and hydrothermal conversion are only used for animal carcass disposal. Different carcass disposal practices are attributed with various environmental problems; the release of leachates containing hazardous chemical compounds, pathogenic organisms, or gaseous emissions, have been identified as major environmental issues. Table 2 summarizes types of pollutants derived from different carcass/corpse disposal technologies, while specific technologies and associated issues are described in detail below.

**Table 2:** Contaminants derived from different carcass and corpse disposal technologies

Disposal method	Major contaminants	Reference
Carcass disposal technologies		
Burial	BOD Ammonia–nitrogen TDS Chloride Alkalinity Sulfate COD Steroid hormones Antibiotics	[13, 32, 46, 156, 157]
Open burning and incineration	Dioxin Polycyclic aromatic hydrocarbons Smoke/ash Heavy metals (Cd, Pb)	[65, 68, 69, 158]
Anaerobic digestion	Pathogens Greenhouse gases	[78]
Alkaline hydrolysis	Alkaline effluents	[79]
Composting	Ammonia–nitrogen TOC	[93]
Corpse disposal technologies		
Burial	Methane Hydrogen sulfide Ammonia Phosphine Volatile amines (putrescine and cadaverine) Mercaptans Heavy metals	[29, 31, 49, 51, 56, 58]
Cremation	Particulate matter Sulfur dioxide Oxides of nitrogen Carbon monoxide Hydrochloric acid Hydrofluoric acid Ammonia VOCs Heavy metals Polychlorinated dibenzo-*p*-dioxins Dibenzofurans	[71, 72, 159]

#### Burial

Land burial is one of the most common and traditional disposal methods for both daily and catastrophic animal mortalities. However, this technique has also been used for corpse disposal resulting from wars or natural catastrophic events. For example, mass corpse burial was extensively utilized during World Wars I and II [29]. Land burial is preferred due to the limited infrastructure requirements, minimal disposal costs, and owners’ convenience [32]. Trench-type burial, landfill-type mass burial, or small on-farm burial is commonly deployed in different areas globally [33]. However, proper site selection, trench design, and site maintenance should be in accordance with the criteria established by relevant authorities to ensure minimum environmental risk. For example, burial should not take place within 350 m of surface water, or any private or public drinking water wells, or within the boundaries of river floodplains [34]. Carcasses should be placed in an approximately 0.9–1.2 m deep compacted trench, and the trench should be covered with mounded soil. Monitoring, together with personnel/equipment safety precautions, should also be required. According to research, carcass burial may have detrimental environmental impacts, resulting in soil, water and air pollution [35, 36]. Thus, carcass land burial is banned in the European Union and in some parts of the USA. In areas where this practice is allowed, strict legislation has been enacted to maintain limitations [14, 36]. Nevertheless, in most cases, burial is still permitted for catastrophic mortality events.

##### Carcass and corpse burial issues

Despite the fact that burial is the most common carcass/corpse disposal method, and is highly applicable when large numbers are involved, only a few sites may provide the appropriate soil and hydraulic properties for environmentally safe burials. The areas situated within close proximity to surface water sources, roads, human settlements and soil associated with shallow groundwater tables have been considered unsuitable for burial sites [37]. There is a high probability for contaminants to enter the surrounding soil, groundwater, or atmosphere when burial pits are made by disregarding the guidelines provided by respective authorities. Several studies have reported soil and groundwater leachate and atmospheric gas contamination associated with carcass burial sites [33, 38, 39]. Negative effects from carcass burial on shallow groundwater have been reported [13]. It has been estimated that resulting groundwater contamination can persist for ~ 20 years due to prolonged carcass decomposition timeframes [33]. According to a study conducted by Watkiss and Smith [39], about 10 years were required to decompose buried livestock carcasses, while the leachate release remained constant for over 20 years [38, 39]. The major reasons for groundwater contamination with releasing leachates have been identified as improper construction of burial pits, damages to liners and rising groundwater tables [37]. Significant greenhouse gas emissions from U.S. animal carcass land burial systems have also been reported [36]. Improperly managed carcass burial sites may also pose a poisoning threat to wildlife and fish populations [33]. Salcedo and Kim [40] found that carcass leachates can act as a point source for generating antibiotic resistant microbial strains. Due to the negative environmental effects, it should be emphasized that while burial places the problem “out-of-sight, out-of-mind”, it does not mean an end to the problem [13].

Site-specific concerns for carcass disposal regarding explicit contaminants are also increasing [13, 32, 41]. Several case studies have detected elevated levels of BOD, ammonia–nitrogen, TDS, and chloride in close proximity to burial sites [13, 42, 43]. Groundwater contamination with ammonia, nitrate, chloride, and fecal pathogens discharged from carcass disposal pits has also been reported [38, 44, 45]. Similarly, McArthur and Milne [46] observed a total of 4,000 m^3^ of leachate generated with elevated BOD, COD, alkalinity, and ammonia–nitrogen from a burial site of FMD carcasses [46]. Elevated BOD (230 mg L^−1^), ammonia–nitrogen (403 mg L^−1^), TDS (1527 mg L^−1^), and chloride (109 mg L^−1^) were detected in groundwater samples collected from an area near disposal pits which contained 28,400 kg turkey carcasses and 6 swine carcasses [47]. Yuan et al. [32] detected steroid hormones, antibiotics, and other nutrients associated with the land burial of cattle carcasses.

Mass human corpse burial induces comparatively similar environment-related hazards to mass animal carcass burial. The specific soils associated with corpse burial sites are called “Necrosols” [48]. Typically, necrosols often become enriched with organic and inorganic nutrients, and have been observed to release volatile gases including ammonia, methane and hydrogen sulfide [49–51]. Decomposition of a human corpse generates approximately 0.4–0.6 L of leachate for each kilogram of body weight, and at a density of 1.23 g cm^−3^ [52] can lead to downward pollutant movement. This leachate is characterized by high BOD, pH, conductivity and chemical compounds including phosphorus, nitrogen, Na^+^, Ca^2+^, Cl^−^, ${\text{HCO}}_{3}^{ - }$, and 10% organic compounds [18, 53]. Necrosols also have the possibility to be contaminated with heavy metals such as Pb, Cr, Zn, As, Mo, and Cu through the decomposition of paints, preservatives and metallic components from coffins [54, 55] Amuno [31] found slightly higher concentrations of 12 trace elements (As, Ba, Cr, Cs, Ga, Ni, Rb, Sc, Th, V, Y, and Zr) in soil of mass grave sites in Rwanda compared to offsite soils. However, that study revealed that the elemental concentrations were not great enough to generate any environment concern. Spongberg and Becks [56] observed elevated heavy metal concentrations (Pb, Cu, Zn, Co, As, and Fe) in a cemetery necrosol situated in Ohio, USA. Moreover, the mass burials used for disposal of soldier corpses typically contain badges, weapons, buttons, etc., that can release heavy metals and other toxic chemicals to the soil and groundwater [29].

Pathogen release to groundwater and surrounding soil is one of the major concerns of carcass/corpse burial. Pathogenic microorganisms including Enterobacteriaceae, Bacilli, Streptococci, Staphylococci, and Clostridia are associated in human tissues, and can act as potential contaminants if released through corpse decay [57, 58]. However, during a pandemic event, potential high risk for spreading virulent microorganisms would be associated with corpse burial regardless of the pathogenic survivability in the soil environment [58]. Favorable environmental conditions such as low temperature, alkalinity, high organic matter and moisture content may increase pathogenic survivability [59]. Prolonged survivability is especially true for spore-forming bacteria species including *Clostridium* sp., which have the ability to survive long time periods even under unfavorable environmental conditions [18, 60]. Abia et al. [61] suggested that corpse burial sites would be a source for microbial pollution in groundwater. The most important bacteria that can pose health risk via transportation with water sources include *Shigella* sp., *Salmonella* sp., *E.coli*, *Yersinia enterocolitica*, *Y. pseudotuberculosis*, *Leptospira* sp., *Dyspepsia coli*, *Francisella tularensis*, *Vibrio* sp., *Pseudomonades*, and *Legionella* sp.; viruses include hepatitis virus, Coxsackie viruses, polio virus, adenovirus, Norwalk-like virus and rotavirus [62]. Because of these reasons, mass land carcass and corpse burial during and after pandemics/epidemics should be carried out with extreme caution to prevent further environmental spread.

#### Open burning and incineration

The open burning of animal carcasses is another commonly used disposal method in many countries. Livestock carcass burning was used during disease epidemics, such as UK FMD outbreak (2001) and the Ugandan anthrax outbreak (2004/2005) [14]. Incineration and cremation are especially suitable for carcass/corpse disposal to prevent further spread of pathogenic organisms resulting from epidemic or pandemic events. For example, cremation has been recommended by China, Sri Lanka, India and USA governments for corpse disposal resulting from the COVID-19 pandemic. Although open burning historically played an important role, now it has become a controversial option for carcass management. In many circumstances, carcass burning should be accomplished in compliance with a waste management license, registered waste exemption, or permit related to pollution control. Furthermore, important considerations such as site location and accessibility, type of animal carcass involved, fuel availability, carcasses quantities to burn, and environmental conditions, should be taken into account [34]. Following burning, it has been recommended that the ashes should be buried and the area should be cleaned, graded, or plowed within 48 h [63].

When open burning, it is not always possible to achieve complete carcass burning, which can lead to increased toxic emissions and infection risks. Thus incineration, where animal carcasses are burnt at high temperatures (≥ 850 °C), is considered to be biologically safer than open burning. Incineration should be carried out in a specific unit operating in compliance with local laws and ordinances [14]. Pyrolysis, gasification, or other thermal treatment processes resulting in an organic ash that is expected to be free of infective agents may be considered in replacement of incineration [14, 64]. Organic ash produced from a typical carcass incinerator represents 1–5% of the input volume, and substantially reduces the environmental footprint associated with carcass disposal [65]. However, incineration gas emissions are a concern, especially under circumstances where the technology may fail to meet environmental standards. Capacity constraints, as well as capital and maintenance costs, are other possible limitations associated with incineration. Carcass moisture content also adds to the energy requirements and costs, thereby making incineration infeasible for routine mortalities. Although incineration is a viable option for the disposal of limited carcass quantities, the cost is insurmountable for large mortalities or catastrophic events [14].

##### Open burning and incineration issues

Open burning or incineration of animal carcasses under inadequate operating conditions or incomplete burning can cause emissions as dioxin, hydrocarbons etc. It has been estimated that dioxins released from animal incineration constituted 0–5% of total dioxin emissions in the UK [66] In addition, a study by Chen et al. [65] calculated the emitted total polycyclic aromatic hydrocarbons (PAH) by carcass incineration during the FMD outbreak in Taiwan in 1997 as 226.2 kg day^−1^.

The ash from open burning can be transported by wind, and not only contaminated soils and waters, but may enter the human food chain. Although the released dose is low, long term exposure to these chemicals have increased the risk of low birth weight and congenital anomaly in babies born close to pyre sites [66, 67]. In Belgium, elevated levels of dioxin, Pb and Cd were found in blood samples from children living close to waste incinerators [68]. Similarly, Dummer et al. [69] found an enhanced risk of lethal congenital anomaly in relation to incinerator proximity. Another negative impact is that burning smoke can be a contributor to climate change [70].

Similarly, corpse cremation is known to produce a range of environmental pollutants including SO_2_, CO, NO_X_, VOCs, NH_3_, polychlorinated dibenzo-*p*-dioxins, dibenzofurans, particulate matter, and heavy metals [71, 72]. In some regions of the world, low chimney height of standard crematories can lead to pollutant dispersal at or near the ground level, potentially affecting human health [73, 74]. Therefore, public concern over cremation is rising in countries like China where cremation is utilized for approximately 47% of corpses [72].

#### Anaerobic digestion

Anaerobic digestion is the degradation of organic material by a mixed culture bacterial ecosystem under anaerobic conditions. Biogas (methane), liquid and solid fertilizers are the final products of anaerobic digestion. In Europe, anaerobic digestion has been successfully used as a viable option for large animal carcass management (e.g., dairy/beef cattle, swine) [75]. Anaerobic digestion produces useful bioenergy in the form of methane [14]. For mesophilic or moderate temperature digestion, a digester temperature of 35 ºC with a retention time of 15–20 days is common. For thermophilic digestion, the digester is adjusted to 55 ºC with a retention time of 12–14 days [76]. However, it has been found that the operational temperature of thermophilic digestion is not high enough to kill pathogens, such as transmissible spongiform encephalopathies [14]. Unfortunately, some researchers suggest that on-site dairy cattle carcass disposal via anaerobic digestion is suitable only for very large operations, due to the significant infrastructure costs [75]. Although anaerobic digestion of large animal mortalities is technically feasible, site-specific variables, such as the economic value of methane production and cost of other available disposal methods, should be considered before implementation.

##### Anaerobic digestion issues

Although anaerobic digestion has been considered as a cost effective and energy-renewable technology, it may not be capable of degrading more resilient pathogens such as *Bacillus anthracis* [77]. According to Maynaud et al. [78], *Campylobacter coli* and *Listeria monocytogenes* can regrow after land application, leading to public health hazards, especially if the digestate is not properly treated with chemical additives or pasteurization prior to or during land application [78]. In addition, intermediate-chemical accumulation, such as volatile fatty acids generated under unstable operating conditions, may lead to environmental toxic effects including acidification and heavy metal accumulation in digested products [79]. Moreover, greenhouse gas production (e.g., methane and carbon dioxide) may occur during anaerobic carcass digestion, contributing to global warming [13].

#### Rendering

Rendering is the process of converting animal carcasses into value-added products, such bone meal and animal fat. It is primarily a heat-driven process by which carcasses are exposed to high temperatures in order to accomplish material physicochemical transformations and pathogen destruction. The traditional rendering method uses pressurized steam in large closed tanks followed by a grinding process. As an alternative to this, a new method of dry cooking the material in its own fat by dry heat in open steam-jacketed drums has been introduced [34]. The temperature and time are commonly selected based on the raw material composition, and are primary determinants of final product quality. Recovered animal fats and proteins are used industrially, or as valuable ingredients to sustain animal agriculture. Rendering is considered one of the most environmentally sound disposal methods, because if properly managed, it facilitates recycling and beneficial reuse of materials that otherwise would be considered waste [34].

##### Rendering related issues

When infrastructure is available, rendering is considered a very effective, convenient, and economical disposal method for carcasses, as well as having the advantage of yielding marketable products. Nevertheless, biosecurity and disease transmission risks are major rendering concerns with the rendering method. Rendered byproducts may also contain transmissible bovine spongiform encephalopathies. Thus, the rendering industry should assume responsibility for maintaining raw material controls, ensuring final product(s) bio-safety, and gaseous or liquid effluent management according to local, regional, or country-wide regulations [80].

#### Alkaline hydrolysis

Alkaline hydrolysis was introduced in the 1990s as a new carcass disposal technology. It is carried out by converting animal carcasses to a sterile aqueous solution of amino acids, sugars, and soaps, using alkali at elevated temperatures [34]. Enzymes, metal salts, acids or bases can be used as catalysts for the hydrolysis process. Among them, alkaline hydrolysis can occur in fixed or mobile facilities, where carcasses are placed in sealed containers with solid or solution type alkali (14). Heat (150 °C) is typically applied and the process is maintained for up to six hours in order to increase degradation processes [13, 14]. The resulting effluent consists of a nutrient rich sterile liquid and bone residue, both of which may be used as soil amendments [13, 81, 82].

By using alkaline hydrolysis, most negative public perceptions associated with carcass disposal can be avoided, as the process inactivates pathogens, sterilizes, and yields environmentally safe materials. Alkaline hydrolysis was the preferred disposal method for bird carcasses infected with avian influenza H5N1 [83]. However, large scale alkaline hydrolysis use may only be feasible for relatively small carcass sizes (birds instead of cattle). The large initial investment cost, expensive maintenance, and limited capacity for large volume carcasses limit the wide use of alkaline hydrolysis for carcass disposal [34].

##### Alkaline hydrolysis related issues

The disinfected solid fraction remaining after alkaline hydrolysis treatment is disposed of in solid waste landfills. However, the liquid effluent generated from this treatment may have a high pH and high organic compound concentrations [79]. Discharge of these effluents into water sources may pose negative environmental impacts. It has been reported that alkaline pH (8.5–10) causes ammonia toxicity to most fish. Further, alkaline leachates may lead to precipitation of calcite [84]; if these precipitates enter water bodies, they can smother aquatic habitats (including benthic and littoral) and reduce light penetration to primary benthic producers [85]. Another significant issue is that trace metals that form oxyanions (e.g. As, Cr, Mo, Se, V) can be highly mobile in alkaline waters, and thereby may pose different environmental and biological threats [86].

#### Other techniques

In addition to the commonly approved disposal methods mentioned above, composting, fermentation, hydrothermal conversion, dry extrusion, refeeding to scavengers, and ocean disposal are other carcass management forms [14, 34]. Although these methods are not applicable for catastrophic events, they are occasionally utilized by the private, agriculture, or business sectors.

Composting is a controlled, aerobic decomposition process that relies on beneficial microorganisms, such as bacteria and fungi. Under optimal conditions, these microbes may decompose carcasses into a humus-like material useful as an organic soil amendment, slow release fertilizer, or water-saving mulch [34]. However, some spore-forming bacteria and prions may persist after composting, presenting a risk to animals grazing in pastures treated with carcass compost. Consequently, many countries have prohibited composting of large or causative animals [77]. Furthermore, although composting is considered an inexpensive, environmentally sound, easy and universal disposal method for biological waste, according to legislation in many countries, composting is not allowed when biosecurity is an issue. Biosecurity agencies in the USA, Australia, New Zealand and Canada have identified the potential for composting as only an emergency livestock mortality management practice [87]. Despite this, an advanced bio-secure composting techniques can still be effectively used for carcass management when the situation requires emergency disposal. This technique utilizes passively aerated composting systems wrapped with plastic to ensure secure composting without environmental release of virulent organisms [88].

Fermentation is a possible method to ensure material stabilization and pathogen reduction. In this method, carcasses are ground to fine particles, mixed with a fermentable carbohydrate source and culture inoculant, and then added to a fermentation container [13]. This allows for carcass preservation until rendering, by using low pH conditions which prevent undesirable degradation processes. The major drawback of fermentation is the high initial investment cost [13].

Hydrothermal conversion may be another promising carcass disposal method, during which animal waste is converted into a liquid product. This liquid is often called bio-oil or bio-crude, created under high-pressure thermochemical process conditions. The advantage of this method has been associated with waste-to-fuel energy recovery as high as 80%. Recently, swine carcasses have been successfully converted into bio-oil that could be upgraded to liquid transport fuels [89]. Furthermore, hydrothermal conversion of animal parts may generate bioactive compounds such as keratins and bioactive peptides that can be used as agricultural fertilizers [90, 91]. However, the strict process conditions and high investment cost pose a significant obstacle to its worldwide use.

Dry extrusion yields usable byproducts, such as feed ingredients, using friction as a means of creating heat, shear, and pressure. This method is also effective enough to inactivate most pathogens, yet the associated high capital cost is a major drawback [92]. Refeeding is another low cost and low-technology carcass disposal option, however palatability must be properly maintained in order to minimize biosecurity concerns [92]. Disposal of carcasses by scavengers and ocean disposal may be categorized as obsolete mortality management options that are not recommended due to a number of environmental, health and social impacts.

##### Issues from other disposal technologies

Proper carcass disposal technologies can effectively reduce the infectious hazards of mortality waste and prevent scavenging. However, these disposal methods still give rise to other health and environmental hazards. For example, significant amounts of contaminants such as ammonium-nitrogen (190–1400 mg L^−1^), TOC (1000–10,000 mg L^−1^), and total solid (5000–30,000 mg L^−1^) have been found in leachate beneath carcass composting units [93] and these contaminants may affect soils and groundwater. Rendering can emit gases and odors, harming the aesthetic value of the environment if not eliminated by properly operated scrubbers [93, 94]. Insufficient pathogenic deactivation during composting may pose a risk to surrounding biodiversity and public health [87]. None of the available disposal techniques are 100% effective in treating carcasses, and all are associated with the risks of soil, water, and air contamination. Hence, a remediation plan should be mandated for all carcass disposal methods.

## Remediation measures

### Soil treatment

To date, techniques such as excavation, washing, soil replacement, biodegradation or bioremediation, phytoremediation, advanced oxidation, and soil vapor extraction, have been applied to remediate sites contaminated with both organic and inorganic pollutants; carcass disposal sites would be no exception [95–98]. Excavation, soil replacement, and soil washing are used when immediate treatment action is required [99]. However, these techniques are often costly, require human resources, and allow for environmental contaminant exposure [100].

The intensification of natural biological degradation processes (i.e., biodegradation or bioremediation) may be preferred as a cost-effective method of removing contaminants from soil environments. Though inorganic compounds are not biodegradable, they can be bio-transformed so that resulting compounds are less toxic or less mobile. Bioremediation relies on proper environmental conditions such as pH, temperature, oxygen, nutrients, and soil moisture [101].

Phytoremediation of contaminants using hyperaccumulator plants is another option for carcass disposal sites. During phytoremediation, plants are used to increase the degradation of organic contaminants, in concert with root rhizosphere microorganisms [97]. Phytoremediation of dredge sediments in landfills can be up to 79% effective in reduction of organic contaminants, mineral oils and polyaromatic hydrocarbons [102]. Plants such as *C. arietinum* and *Vetiver* show promise for absorbing antibiotics, and thus may have the potential to remediate antibiotic-contaminated carcass disposal sites [96, 97]. However, phytoremediation effectiveness depends on the disposal site contaminant, the environment, and the plant species.

Some advanced techniques, such as oxidation processes, are relatively expensive, and by-products generated during oxidation may be more resistant to degradation [103]. Soil vapor extraction is an accepted method for the remediation of volatile organic compounds in soils. This cost-effective method allows for biodegradation to fewer volatile compounds through increased airflow. Unfortunately, soil vapor extraction is not appropriate for contaminated sites with shallow water tables (< 0.9 m from the surface) due to occurrence of upwelling that inhibits soil vapor flow [104].

### Water treatment

When water contamination occurs due to carcass disposal issues, satisfactory treatment is not an easy task. Water contamination typically occurs due to the presence of organic pollutants, nutrients, and toxic compounds, as well as pathogenic microorganisms. The remediation technology choice depends largely on contaminated water characteristics, discharge limitations, and site constraints. Suggested clean-up methodologies include the use of chemicals, advanced oxidative processes, biological approaches, use of wetlands, emerging technologies, and adsorbents.

Alkaline chemicals (e.g., lime) can be used to inactivate pathogenic microorganisms in contaminated water. Disadvantages of this approach are potentially large odor and ammonia emissions, high cost, and further toxic environmental effects [105]. A number of advanced oxidation processes, including photocatalysis, Fenton, photo-Fenton, and dielectric barrier discharge plasma systems, have been suggested as viable options to remove aqueous contaminants such as pharmaceuticals [106–109]. These techniques are highlighted as energy efficient, cost-effective, and environmentally friendly processes that can be used in large-scale water treatment [109].

Among the biological methods, aerobic processes are commonly used for organically-contaminated water treatment due to its relatively high efficiency [110]. Anaerobic treatment is another remediation option for fully-degradable substances in carcass-contaminated waters [111]. Moreover, anaerobic techniques have been proven to significantly reduce COD and BOD in contaminated waters [112]. In addition, constructed wetlands are land-based water treatment systems designed for treating wastewaters and contaminated waters [113]. They have been widely employed for livestock wastewater treatments because they are compatible with typical farm and ranch operations. Further, they are mostly effective in reducing micro contaminant concentrations, including antibiotics residues [114, 115]. Alvarez et al. [116] used choline dihydrogen phosphate (ChDHP), a biocompatible ionic liquid, to treat swine wastewater streams. This emerging technology has been proven, promising option for swine effluent antibiotic removal [116]. The above approaches may have some potential use for contaminated water remediation resulting from animal carcass and human corpse disposal sites.

Adsorption as a remediation tool is a fast, inexpensive, simple, and universal method, compared to other physical treatment technologies (e.g., sedimentation, flotation, ion exchange, and reverse osmosis). Adsorption efficiency largely depends on the adsorbent used. Water solution chemistry also plays an important role in the remediation/adsorption process [117]. Adsorbents, such as clay minerals [118], biomaterials [119], and zeolites [120] have been widely used to adsorb ions and organics in contaminated water. Activated carbons are also common and widely used adsorbents for the removal of inorganic and organic impurities, as well as undesirable odor and color, in wastewater. The adsorption capacity of activated carbon is attributed to its large surface area, microporous structure, variable surface characteristics, and high degree of surface reactivity [121]. Activated carbon has also been successfully used for the remediation of refractory organic compounds like tetracycline and sulfamethoxazole, which may persist in carcass leachate [122]. However, high production and operation costs, regeneration issues, and difficulty in operation are the potential drawbacks of using activated carbon for contaminant adsorption. Other means of adsorption via alternative carbon sources, could be an attractive means for carcass disposal reclamation scenarios.

### Biochar for managing mass carcass and corpse disposal sites

In the search for suitable absorbent alternatives, many researchers have demonstrated that biochar is economically attractive, and may act similar to or even outperform activated carbon [123]. Biochar is the thermal degradation product of various materials or carbon-based products that otherwise would go to waste. Thus, biochar technology can enhance the environmental value of waste by reducing disposal cost and potentially improving sustainability via use in reclamation efforts. Biochar may be used for removing both soil and water contaminants due to the presence of oxygenated functional groups, increased cation exchange capacity, large surface area, high porosity, and the presence of oxides, hydroxides, and carbonate phases [124–127].

#### Biochar as a sorbent

The specific sorption properties of biochar for various contaminants may largely depend on the pyrolysis temperature, residence time, and feedstock type. Despite the ability to adsorb aqueous and soil phase contaminants, biochar application has shown the potential for reducing various organic and inorganic compounds in soil, as well as being effective in reducing greenhouse gas emissions [128]. Table 3 summarizes the use of biochar, derived from different feedstock materials under different pyrolysis conditions, for the remediation of aqueous, soil, and gaseous phase contaminants.

**Table 3:** Biochar utilization for environmental contaminant removal

Pollutant	Media/conditions	Removal capacity	Partition coefficient	Type of biochar	Production conditions	Reference
COD	water/laboratory conditions	~ 70 mg g^−1^	~ 0.058 L g^−1^	Wood derived biochar	1000 °C	[160]
Ammonium nitrogen	water/laboratory conditions	3.4 mg g^−1^	0.017 L g^−1^	Rice husk biochar Cacao shell and corn biochar	700 °C 300–350 °C	[161, 162]
		44.64 ± 0.602 mg g^−1^	0.032 L g^−1^	Wood biochar	600 °C	[21]
		39.8 ± 0.540 mg g^−1^	0.028 L g^−1^	Rice husk biochar	600 °C	[21]
		0.753 mg g^−1^	0.025 L g^−1^	Corn straw biochar	< 700 °C	[163]
		1.003 mg g^−1^	0.033 L g^−1^	Peanut shell biochar	< 700 °C	[163]
		5.3 mg g^−1^	0.007 L g^−1^	Mixed hardwood biochar	300 °C	[164]
		17.6 mg g^−1^	0.074 L g^−1^	Plant (*Thaliadealbata*) biomass derived biochar	700 °C	[165]
		5.6 mg g^−1^	0.062 L g^−1^	Pineapple peel biochar	300 °C	[166]
		4.71 mg g^−1^	0.069 L g^−1^	Orange peel biochar	300 °C	[166]
		2.65 mg g^−1^	0.034 L g^−1^	Pitaya peel biochar	400 °C	[166]
Antibiotics						
Tylosin	Soil/laboratory conditions	–	–	Pulpgrade hardwood and softwood chips biochar	850 and 900 °C	[167]
Sulfamethazine	Water/laboratory conditions	33.81 mg g^−1^	1.738 L g^−1^	Tea waste biochar	700 °C Steam activated	[135]
	Water/laboratory conditions	37.7 mg g^−1^	1.809 L g^−1^	Invasive plant biochar	700 °C Steam activated	[168]
Tetracycline	Water/laboratory conditions	14.3 mg g^−1^	0.152 L g^−1^	Rice husk biochar	450–500 °C	[169]
	Water/laboratory conditions	101 mg g^−1^	3.061 L g^−1^	Pinewood sawdust biochar	700 °C Pyrolyzed with 1:5, air to NO_2_	[170]
	Water/laboratory conditions	2.5 mg g^−1^	0.138 L g^−1^	Bamboo sawdust biochar	300 °C	[171]
	Water/laboratory conditions	4.4 mg g^−1^	0.267 L g^−1^	Bamboo sawdust biochar	450 °C	[171]
	Water/laboratory conditions	147.9 mg g^−1^	1.623 L g^−1^	*Spirulina* sp. (microalgae) biochar	750 °C	[172]
Sulfamethoxazole	Real wastewater solution	27.7 mg g^−1^	2.815 L g^−1^	Ball milled hickory chips biochar	450 °C	[173]
Sulfadiazine	Water/laboratory conditions	123.0 mg g^−1^	4.415 L g^−1^	Pinewood sawdust biochar	700 °C Pyrolyzed with 1:5, air to NO_2_	[170]
Sulfapyridine	Real wastewater solution	58.6 mg g^−1^	7.600 L g^−1^	Ball milled hickory chips biochar	450 °C	[173]
Hormones						
Estrone	Soil/laboratory conditions	0.035 mg g^−1^	0.097 L g^−1^	Pine saw dust biochar	700 °C Steam activated	[136]
Oestradiol	Soil/laboratory conditions	0.026 mg g^−1^	0.041 L g^−1^	Pine saw dust biochar	700 °C Steam activated	[136]
Greenhouse Gases Methane		0.05–0.9 mol kg^−1^ –	– –	Wood biochar Wheat straw biochar	350–600 °C 350–550 °C	[174, 175]
Carbon dioxide	–	–	–	Wheat straw biochar	350–550 °C	[175]
	–	–	–	Mixed sawdust biochar	500 °C	[176]
Nitrous oxide	–	–	–	Rice husk biochar	450 °C	[177]
	–	–	–	Hardwood	450–500 °C	[178]
	Soil/laboratory conditions	–	–	Pine sawdust biochar	300 and 550 °C	[179]
Volatile organic compounds (VOCs)						
Benzene	Laboratory conditions	54.6 mg g^−1^	–	Neem biochar	550 °C	[180]
Toluene	Laboratory conditions	65.5 mg g^−1^	–	Neem biochar	550 °C	[180]
Methyl chloride	Laboratory conditions	39.6 mg g^−1^	–	Neem biochar	550 °C	[180]
Xylene	Laboratory conditions	60.2 mg g^−1^	–	Neem biochar	550 °C	[180]
Chloroform	Laboratory conditions	30.8 mg g^−1^	–	Neem biochar	550 °C	[180]
Carbon tetrachloride	Laboratory conditions	41.0 mg g^−1^	–	Neem biochar	550 °C	[180]

**Fig. 1:**
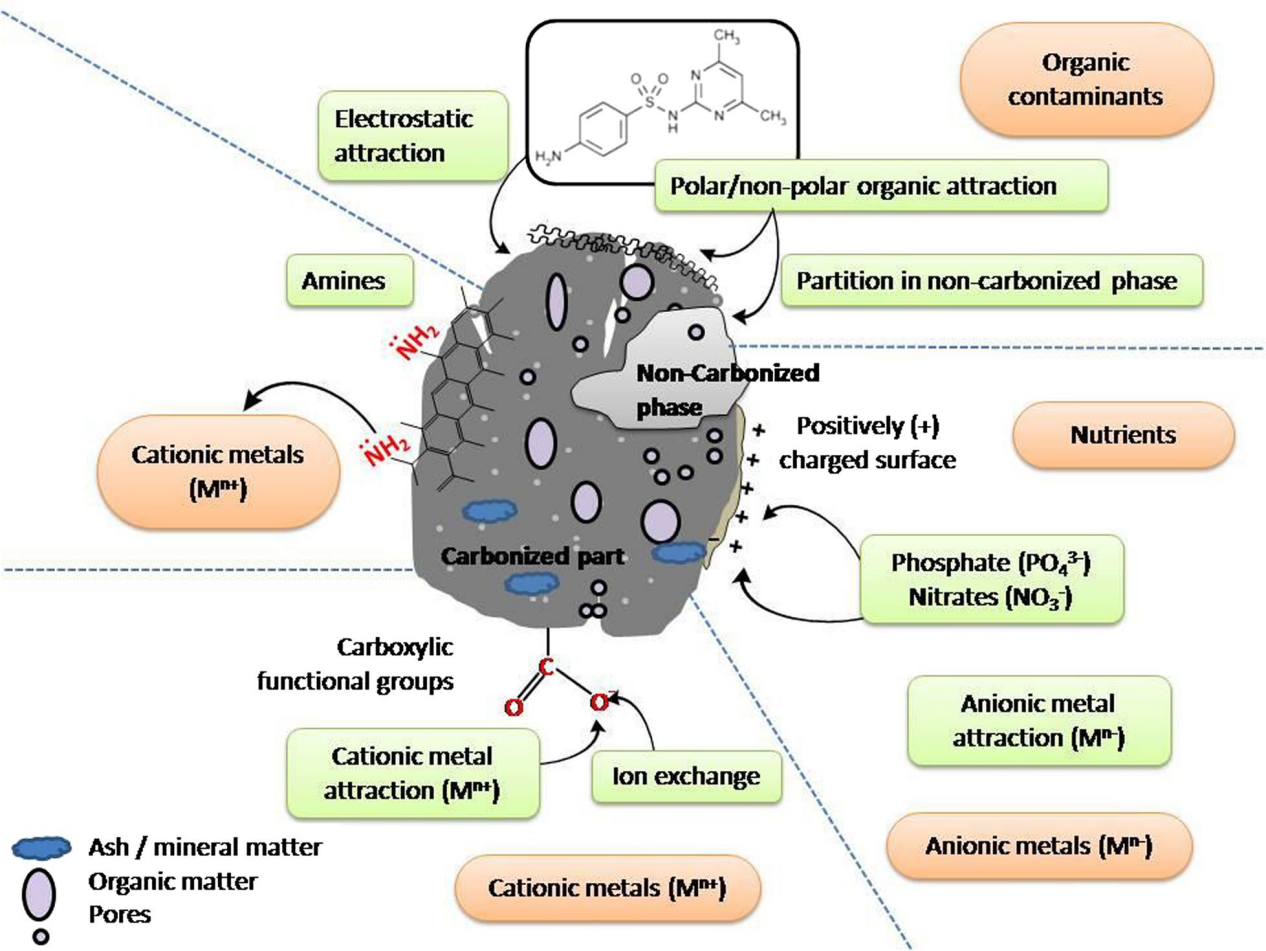
Mechanisms involved in remediating contaminants from carcass disposal sites

Biochar may reduce a variety of mobile organic and inorganic contaminants present in soils. Several mechanisms have been suggested for different contaminant remediation by biochar for anionic metals, cationic metals, and organics (Fig. 1). Several recent studies have revealed that biochar derived from rice husk could significantly adsorb ammonium-nitrogen and phosphate [129, 130]. Ammonium-nitrogen adsorption capacity of rice husk biochar was found to increase with decreasing biochar particle size [21]. It has been suggested that smaller sized biochar particles may lead to decrease hydraulic conductivity, extend the contact time with pollutants, and thereby enhanced remediation efficiency [21, 131].

**Fig. 2:**
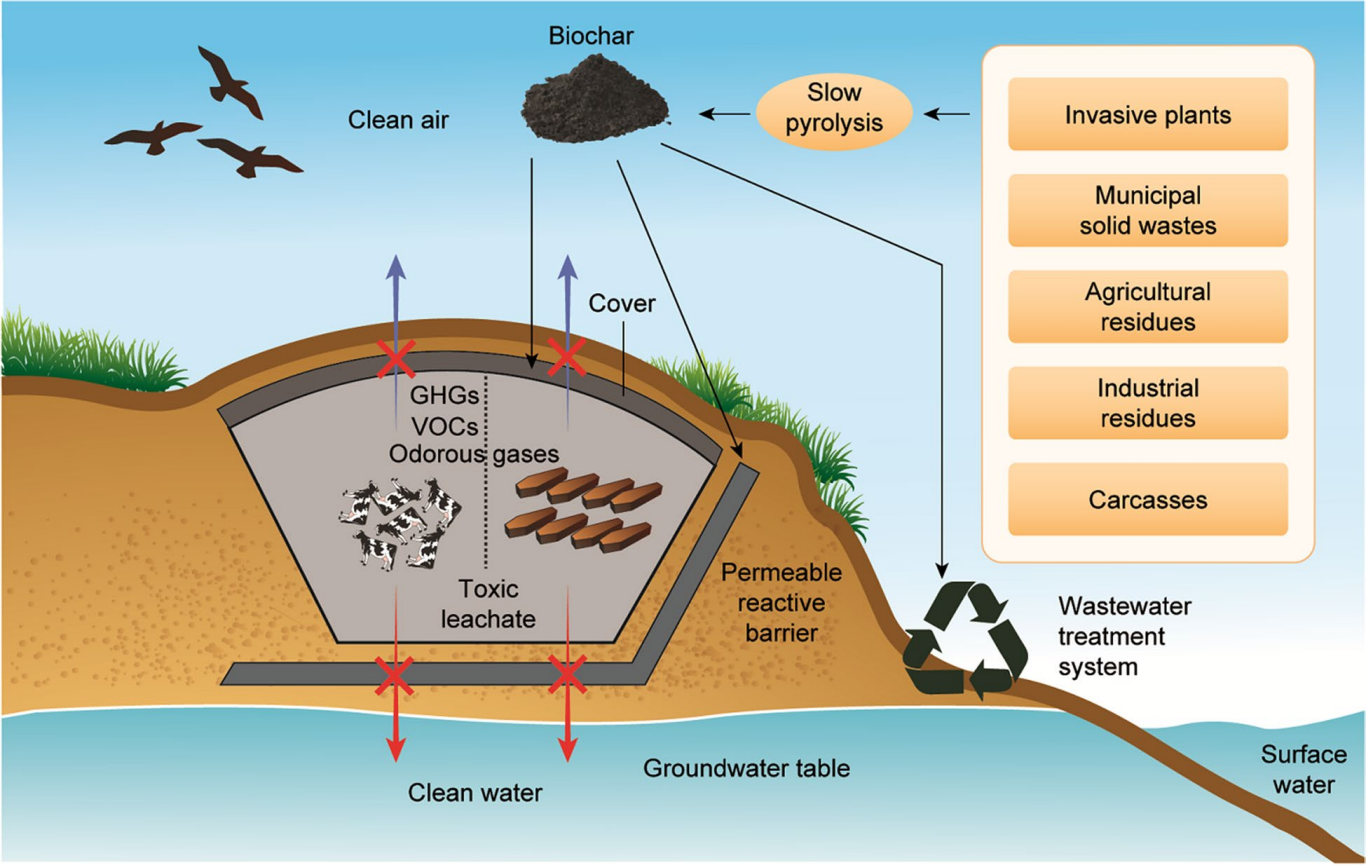
Various applications of biochar in mass burial sites

Anionic metals/metalloids may be complexed with positively charged sites on biochar surfaces, depending on pH and pH at the zero point charge of the media [132]. At the same time, cationic metals in carcass leachate may be removed by various mechanisms such as ion exchange, surface complexation, precipitation (as oxides, hydroxides or carbonates), and reaction with amines on biochar surfaces (Fig. 1). In terms of organic contaminants, π–π electron donor–acceptor interaction, polar and nonpolar interactions, electrostatic attraction, and partition into the non-carbonized biochar phase have been reported [124, 133, 134]. Moreover, organic contaminants can be remediated through pore diffusion and adsorption on interior biochar surfaces. Sulfonamide, a veterinary pharmaceutical which may enter the environment through carcass burial site leachate, has been effectively remediated by using steam activated tea waste biochar [135]. The sorption mechanism was explained by physical surface area and pore volume changes that occurred during steam activation, which enhanced sulfonamide diffusion into biochar pores. Similarly, steam gasified pine sawdust biochar showed a high sorption capacity for steroid hormones (oestradiol and estrone), which may be added to soil through livestock faeces and urine deposition [136]. The authors highlighted that biochar created at a relatively high temperature (700 °C) had the appropriate surface area, low ash content and abundant polar functional groups that resulted in a high hormone sorption capacity as compared to lower temperature biochars. Sorption of the antibiotic lincomycin (widely used to control diseases in pigs, dairy cattle and sheep) was examined using manure derived biochar [137]. Relatively quick antibiotic sorption was explained by non-electrostatic interactions including hydrophobic partitioning, π–π electron donor–acceptor interactions, and hydrogen and van der Waals bonding. In addition, biochar macro- and nano-porous structures contributed to long-term lincomycin sorption via slow diffusion into biochar pores. Results implied that biochar could be used as a long-term strategy for antibiotic immobilization in agroecosystems, and likely could be used at carcass disposal sites [137]. Rice husk biochar, modified by methanol, has been shown to sorb tetracycline (a common veterinary drug) [70]. Biochar functional group alteration, due to methanol modification, resulted in the formation of hydrogen bonds between contaminant molecules and biochar, likely leading to enhanced sorption. Biochar use for contaminant sorption and immobilization may be successfully incorporated into an *in-situ* remediation technology for groundwater, known as permeable reactive barriers (PRBs) (96). Kim et al. [129] introduced a PRB containing rice husk biochar for ground/surface water contaminated with swine leachate [129]. Biochar effectively removed ammonium-nitrogen and potassium compared to phosphate, due to its high affinity for positively charged contaminants. The authors concluded that biochar derived from MgCl_2_ or ZnCl_2_ enriched feedstock, or modified base-enriched biochar, could be used to remediate both positively and negatively charged contaminants. Yoon et al. [19] also investigated rice husk biochar use in PRB to mitigate groundwater pollution from a pig carcass burial site, finding that leachate pH, EC, and ammonium-nitrogen were reduced by biochar application as compared to pre-treatment. Biochar also has use as a potential filtering agent for pathogenic organism removal from carcass/corpse leachate or from affected groundwater. Sasidharan et al. [138] demonstrated relatively high bacteria retention in biochar-amended sand columns. A similar study by Kranner et al. [139] found improved removal of the faecal indicator bacteria *Escherichia coli*, enterococci, and male-specific coliphage by biofilters amended with biochar as compared to sand biofilters alone. Thus, in situ biochar application to carcass/corpse contaminated sites may likely be a sustainable strategy for remediation (Fig. 2).

## Carcass derived biochar

**Fig. 3:**
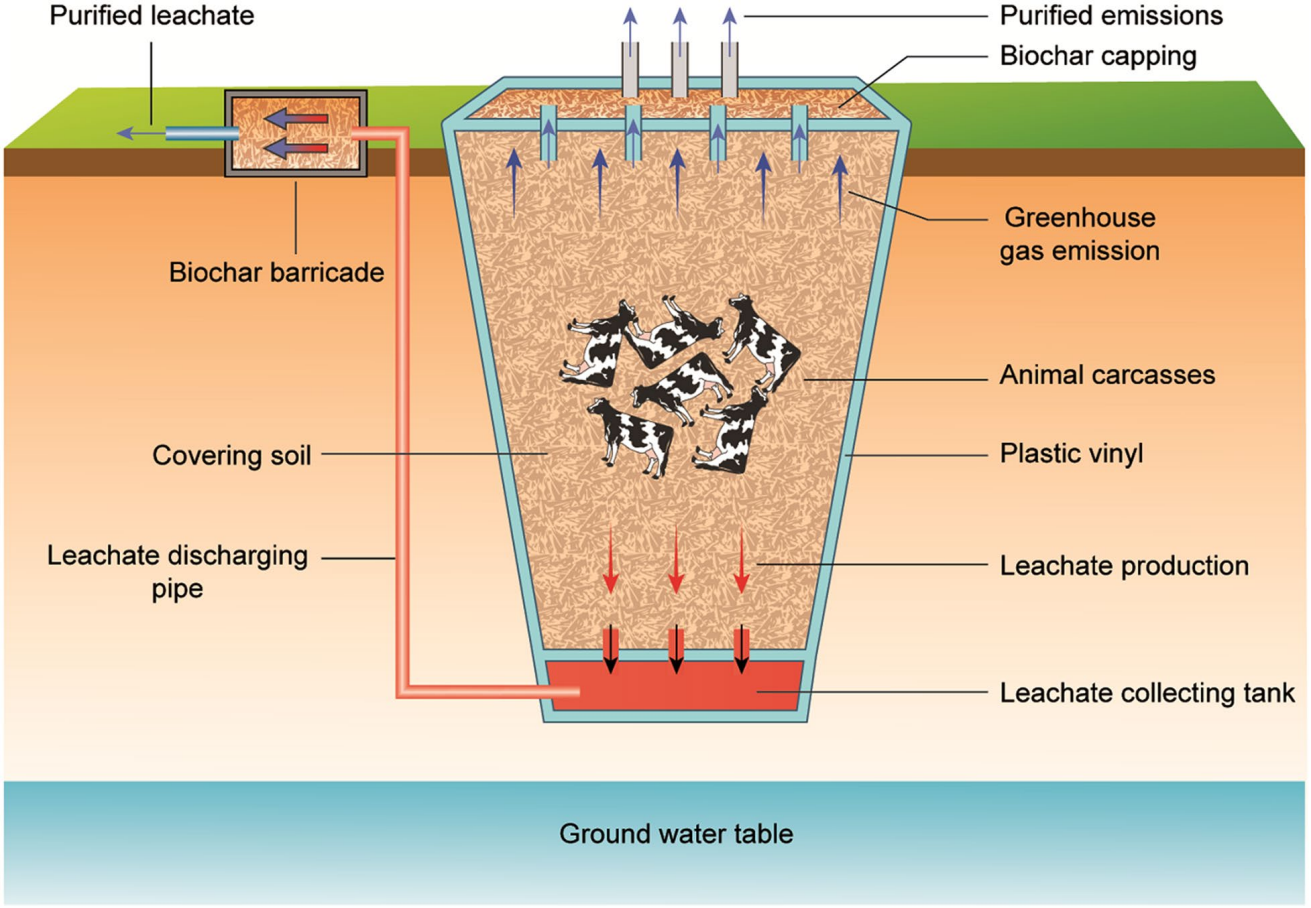
Proposed scheme for biochar utilization from leachate and emissions originating from burial pits (Adapted from [8])

Pyrolytic conversion of carcasses into biochar has been considered an environmentally friendly and economical approach for carcass management [140]. This technique involves less emissions as compared to other management options, while the resulting biochar is considered a potential pollutant sorption material or an effective soil conditioner (Fig. 3). Betts et al. [141] emphasized this contention, noting that meat and bone meal derived biochar had a relatively high sorptive capacity for aqueous Zn. Lei et al. [142] utilized cattle carcass derived biochar to effectively sorb aqueous Cd. Furthermore, the oil produced during carcass pyrolysis may be utilized as a raw material for bio-diesel production [143].

### Carcass-derived biochar as a soil amendment

Animal waste has historically been the primary source of plant nutrients and as an amendment for agricultural crop production. Nevertheless, carcass waste used directly as a soil amendment may lead to disease dispersion risk. Thus, converting carcass waste into biochar via pyrolysis may be a sustainable management option, since heat treatment below typical pyrolysis temperatures (e.g., 500 °C) has proven to be effective at killing associated pathogenic organisms [144]. Animal carcass biochar may provide essential plant nutrients for crop growth. Animal carcass by-products may contain up to 30% P_2_O_5_, which may make it an excellent P source. In support of this concept, Ma and Matsunaka [145] reported that dairy cattle carcass-derived biochar was an effective P fertilizer under acidic soil conditions. Several authors have reported that animal-origin biochar can be enriched in both P and N [146, 147]. A recent study compared pig carcass- to wood-derived biochar (*Platanus orientalis*) for nutrient bioavailability, enzymatic activity, and plant growth performance in soil contaminated with metal-phthalic acid ester; results proved that carcass-derived biochar increased soil P and K availability, urease and acid phosphatase activities, and pak choi yield as compared to wood-derived biochar [148]. Favorable carcass-derived biochar properties include alkaline pH, elevated cation exchange capacity and nutrient content, all of which could benefit soils that either lack nutrients or the ability to retain nutrients. Carcass-derived biochars may also act like other biochars used as a soil amendment. Biochar land application can enhance water infiltration, water holding capacity, soil structural stability, and soil biological activity. It is yet to be seen if carcass-derived biochars will act similar once applied to soils, and therefore carcass-derived biochars should be further evaluated under field conditions.

## Future Perspectives

This review article provides a detailed overview of carcass and corpse disposal techniques, their environmental impacts, and potential remediation strategies. Managing routine or catastrophic mortality is multifaceted, and depends on the cause of death, type, and quantity of animal carcasses or human corpses, as well on location and economic status. The true challenge involves selecting cost-effective and efficient technologies that protect public health and safety, avoid adverse water and air quality effects, and prevent the spread of disease.

Existing techniques used for carcass/corpse disposal include burying, burning, incineration, composting, rendering, and alkaline hydrolysis. Each treatment strategy has both benefits and disadvantages. Burial is the most common carcass and corpse disposal method; however, it can lead to soil and groundwater pollution. During disease outbreak events, burning and incineration are preferred, yet incineration can be costly due to the fuel and operational requirements. Rendering is an emerging technology which converts animal tissues into animal fat or protein for agricultural or industrial use. However, it remains unclear how to properly monitor and control the conditions during rendering in order to maintain biosecurity and reduce disease transmission. Alkaline hydrolysis, is in limited use due to high initial and operational costs, however this process has the potential for completely destroying infectious agents by solubilization and digestion. Other potential methods for carcass management are anaerobic digestion, fermentation, and dry extrusion, although their effectiveness has not been completely proven.

 Although the existing literature focuses on the environmental impacts of carcass waste, there is limited research on remediation. Surface- and ground-water or soil contamination issues are frequently related to carcass disposal; evidence of elevated BOD, COD, TDS, ammonia–nitrogen, antibiotics, and steroid hormones from carcass disposal exist and should be addressed. Many conventional remediation methods are costly and ineffective for large-scale applications. However, adsorption techniques are generally effective and simple, and there is a wide scope of research on the adsorptive removal of organic and inorganic pollutants.

However, research to date has not focused on the treatment of complex carcass leachate; instead, the adsorption of single pollutants has been studied. Furthermore, growing research focus has been directed at finding acceptable, low cost, and efficient alternatives to conventionally utilized activated carbon. Carcass/corpse sites and wastewater remediation using biochar remains a completely unexplored area of study. While previous research has demonstrated that biochar is effective at removing BOD, COD, TDS, nutrients, and refractory chemicals like antibiotics in the environment, it is yet to be evaluated or proven effective in the presence of real-world carcass leachates.

In addition to the effectiveness of biochar at removing a multitude of constituents, it is also promising as a sorbent for methane and other greenhouse gases. Thus, biochar could potentially be used as a bio-cover and might be a suitable material for PRBs. Figure 3 suggests the potential use of biochar in order to treat toxic leachates and gaseous emissions from burial pits. The same approach has broad applicability for minimizing adverse environmental effects following animal/human burial events. Furthermore, biochar derived from animal waste can be enriched in nutrients, leading to improved crop growth. Thus, biochar produced from animal carcasses may be a “win–win” as it can be used for pollutant remediation and serve as a tool to improve soil properties. Creating biochar from and within carcass disposal systems may be worth future investigation.

## Data Availability

Not applicable.

## Abbreviations

LSU: Livestock units
FMD: Foot-and-mouth disease
BOD: Biological oxygen demand
COD: Chemical oxygen demand
TOC: Total organic carbon
TDS: Total dissolved solids
PAH: Polycyclic aromatic hydrocarbons
ChDHP: Choline dihydrogen phosphate
PRBs: Permeable reactive barriers
